# Cocaine in Croup

**Published:** 1887-12

**Authors:** 


**Cocaine in Croup.**—Labric praises cocaine as the best remedy for croup. He applies a brush dipped in a five per cent. solution of cocaine to the throat for several seconds; a few drops are allowed to go down into the larynx. The operation is repeated two or three times a day, and nothing but a little black coffee is administered to the patient.—*N. E. Medical Monthly.*

